# Chronic pain in adults with osteogenesis imperfecta and its relationship to appraisal, coping, and quality of life: A cross-sectional study

**DOI:** 10.1097/MD.0000000000030256

**Published:** 2022-10-07

**Authors:** Rubén Muñoz Cortés, José Francisco Soriano Pastor, Vicente Monsalve Dolz

**Affiliations:** a Fundación AHUCE, Valencia, Spain; b Department of Personality, Evaluation and Psychological Treatments, University of Valencia, Valencia, Spain; c Multidisciplinary Pain Treatment Unit, General University Hospital of Valencia, València, Spain.

**Keywords:** chronic pain, coping strategies, osteogenesis imperfecta, quality of life, threat appraisal

## Abstract

Chronic pain is a common experience in osteogenesis imperfecta (OI). However, there are few studies on this topic, and none of them emerge from psychology as a discipline. The purpose of this work is to describe the frequency of chronic pain and its characteristics in a large sample of adults with OI, as well as its relationship with clinical, sociodemographic, psychological, and quality of life variables.

A cross-sectional study was carried out in a sample of 418 adults with OI who answered a battery of online questionnaires. Sociodemographic and clinical variables, pain parameters, participants’ appraisal of pain, coping strategies, interference in daily activities, and health-related quality of life were evaluated. A descriptive and correlational analysis was performed.

Up to 83% of the sample reported experiencing pain frequently. Both the frequency and intensity of pain were related to the accumulation of fractures over the years (*P* < .05), but were independent of other variables like the severity of the pathology or the use of bisphosphonates. Higher threat appraisal of pain was associated with an increase in perceived pain intensity and its interference with daily activities, as well as a decrease in physical and mental health (*P* < .001).

Chronic pain frequent condition in adults with OI, regardless of the severity of the pathology. It interferes with their usual activities and has an impact on their quality of life. The way in which participants appraise their pain also have an influence on its intensity and its consequences. Interventions aimed at training strategies for managing appraisals about pain could potentially improve adaptation to chronic pain.

## 1. Introduction

Osteogenesis imperfecta (OI), known as brittle bone disease, refers to a heterogeneous group of hereditary bone dysplasias that affect the connective tissue and are mainly characterized by more fragile bones.^[1]^ In addition to the higher likelihood of bone fracture, other clinical signs are common, such as blue sclera, dentinogenesis imperfecta, lax skin, joint hypermobility, short stature and deformities of the long bones.^[2]^ Depending on the severity of the pathology, OI is classified into 5 types^[3,4]^: nondeforming with blue sclera or type 1 is the commonest form of OI, also considered the mildest, with a lower rate of bone fractures and deformities; common variable OI or type 4, of highly variable severity, presents recurrent fractures and osteoporosis, and varying degrees of bone deformities and scoliosis; progressively deforming or type 3, a very severe variant with multiple fractures causing skeletal deformities. Stature is usually short and hearing difficulties and cardiorespiratory complications are more common than in less severe forms; perinatally lethal OI syndromes or type 2, an extremely severe form of OI that causes death in one-fifth of cases during gestation and in 90% within the first 4 weeks after birth; with calcification in interosseous membranes or type 5, featuring moderate to severe bone fragility and greater propensity to developing hyperplasic callus after bone fracture. Prevalence is in the region of 1 case in 10,000 births, no gender-based differences being observed.^[5]^

One of the least studied aspects of this pathology is chronic pain, although the few studies conducted on this subject attest that it is frequent in OI. Indeed, in the context of infancy and adolescence, it has been observed that pain that is not deriving from fractures or lesions is frequently reported, and interferes with daily activity, especially in the case of patients who primarily resort to coping strategies based on prevention.^[6,7]^ Although medication with biphosphonates seems to reduce pain and improve affected patients’ physical functionality,^[8,9]^ this effect has not been confirmed in all studies.^[6,10]^ With regard to adults, still fewer studies are available. Nevertheless, a high incidence of chronic pain has been detected in this collective,^[11,12]^ which disrupts everyday activities and is found to be resistant to surgical, pharmacological and nonpharmacological intervention.^[13]^

The International Association for the Study of Pain (IASP) defines pain as “An unpleasant sensory and emotional experience associated with, or resembling that associated with, actual or potential tissue damage,”^[14]^ mentioning in its “key notes” the subjective nature of this experience, and how this is influenced by psychological and learned factors. Chronic pain, in more specific terms, is that which persists beyond the healing period (generally more than 3 to 6 months) and, therefore, has lost its adaptive value.^[15]^ Based on the transactional model of Lazarus & Folkman,^[16]^ chronic pain can be considered a powerful source of stress involving elements that can be explained from the field of psychology.^[17,18]^ For instance, appraisal defined as “the processes that determine the meaning attributed to pain by an individual”^[19]^ has been associated with the perception of its intensity and unpleasantness,^[20]^ with avoidance behaviors as a response to stimuli appraised as potentially painful,^[21]^ or with lower tolerance toward pain.^[22]^ Similarly, the appraisal of pain influences the coping strategies deployed,^[23,24]^ described as “cognitive and behavioral efforts undergoing constant change that are made to handle specific external and/or internal demands that are appraised as exceeding or overburdening the individual’s resources.”^[16]^ In turn, these are associated with the manner in which each patient adapts to the pain experienced^[25,26]^ and the quality of life reported.^[27,28]^

There is a close link between chronic pain and quality of life, defined by the World Health Organization as *“*individuals’ perception of their situation in life, within the context of their culture and values, and in relation to their aims, expectations, values and interests.” This is a complex, multidimensional concept that encompasses different areas, such as the economic, educational, social, spiritual, or physical and psychological health.^[29]^ As pointed out in the ITACA (Impact of Analgesic Treatment on Quality of Life in Algies) study, conducted at 100 pain units in Spain,^[30]^ a direct relationship exists between the intensity of pain and its impact on quality of life, especially the physical health index. Thus, patients suffering chronic pain perceive that their everyday activities are restricted and report a poorer general health status.^[31,32]^

A large number of studies have assessed quality of life in OI, but the greater part of these were conducted with children. Minors with OI usually report reduced physical health in comparison with the general public, which worsens as the severity of the condition increases.^[33]^ Thus, daily activity and participation in physical activities with peers is hindered by compromised functionality, breathing difficulties, scoliosis, or the risk of further fractures.^[34]^ Similarly, pain has proved to be an important factor affecting physical, emotional and psychosocial aspects in children and adolescents with OI,^[35]^ and is identified as one of the 6 factors with the greatest weight in their quality of life, the others being sense of security, isolation, independence, fear of new fractures, and reduced functionality.^[36]^ As for mental health and the social, academic and emotional areas, scores were similar to those of the general population,^[33]^ although this observation is not corroborated in all explorations.^[35,37]^ In studies targeting adult populations, likewise, mental health indices are analogous to those described for the general population, whereas physical health is reduced.^[33,38]^ Complications deriving from this pathology such as pain, cramps, scoliosis, short stature or bone fragility hinder everyday tasks, contributing to a dimmer perception of physical health, especially in the most severe cases.^[34,39,40]^ Nevertheless, despite the drawbacks and limitations to activity, adults with OI usually report a high level of satisfaction with life.^[12]^

Despite the notable presence of chronic pain among adults with OI, and its fundamental relationship with quality of life, only a few studies have focused on this topic. Likewise, none of the existing studies have emerged from the discipline of psychology, due to which the variables that are significant in configuring the experience of pain were not taken into account. Similarly, studies commonly involve small samples, due mainly to the low prevalence of this pathology. This study aims to assess the presence of chronic pain, its characteristics and the relationships it establishes with clinical, sociodemographic, and psychological variables, such as pain appraisal and coping strategies, and quality of life variables in a wide sample of adults with OI.

## 2. Materials and Methods

### 2.1. Sample

The sample consists of 418 adults with OI of different nationalities. Participants, having granted their consent, individually answered an online battery of questions designed to evaluate variables related to chronic pain, sociodemographic and clinical data, psychological aspects such as pain appraisal and coping strategies, the interference of pain in daily activities and quality of life.

The inclusion criteria for participants were as follows:

1. Be at least 18 years of age
2. Having an OI diagnosis
3. Having access to the internet

The exclusion criteria were as follows:

1. Being under 18 years of age
2. Not having an OI diagnosis

### 2.2. Procedure

To meet the objective of gathering a broad sample, a survey was set up online on a website server. This was configured in 2 languages, Spanish and English, requiring the translation of questionnaires that were not available in either of these. For this task, a double translation process was applied followed by statistical validation. Each participant was free to complete the questionnaire from a computer, mobile phone, or tablet, individually and without the presence of an evaluator. The time taken to answer the questions was approximately 20 minutes and the information was stored on a website server accessed exclusively by Fundación AHUCE (Spanish Osteogenesis Imperfecta Foundation). All data were anonymous.

The online survey was distributed throughout Spain and abroad among the target population. National distribution was conducted by Fundación AHUCE, the Asociación Nacional Huesos de Cristal (AHUCE), and Asociación Madrileña de Osteogénesis Imperfecta (AMOI), using various channels such as social networks, e-mail, information leaflets and communications at meetings and conferences. International distribution was run by the Brittle Bone Society in the United Kingdom, the Osteogenesis Imperfecta Foundation (OIF) in the United States, the European Federation of Osteogenesis Imperfecta (OIFE), and other organizations in other countries. The distribution channels were chiefly social networks, e-mail and electronic journals. The survey remained open from June 15, 2018, to January 15, 2019.

### 2.3. Evaluation instruments

#### 2.3.1. Clinical and sociodemographic variables.

The sociodemographic questionnaire, drawn up by the research team, consists of 7 questions through which the following variables are registered: age, gender, country of residence, marital status (single, married, partnership, widowed, or divorced), residence status (living alone, with parents, with my partner or family, other situation), education level (uneducated, primary school, secondary school, vocational training, university degree or other), and job status (studying, working, unemployed, pensioner of working age or retired). The format used for answers varied, combining open and multiple-choice questions.

Similarly, the clinical questionnaire was drafted by the research team in collaboration with health professionals experienced in OI, and subsequently reviewed and approved by the scientific committee of Fundación AHUCE. This document contains 12 items of clinical interest to this pathology, in which the questions explore the date of diagnosis, age at which the first fracture occurred, the approximate number of fractures to date, visible clinical signs (short stature, blue sclera, hearing loss, vertebral collapse, eyesight problems, hypermobility of the joints, and frequent sprains), the existence of a genetic study and the affected gene, degree of severity (mild, moderate or severe, based on the Vann Dijk and Sillence’s grading scale proposal),^[3]^ OI type, the use of orthopedic aids (walking frame, crutches, wheelchair, other aid, and no aid), the type of medical treatment received in the last 2 years, the frequency of physical exercise (none, once a week, twice, or more per week) and the regularity of visits to the physiotherapist (never, once a month, twice per month, and at least once a week).

#### 2.3.2. Chronic pain and its characteristics.

The frequency of pain is assessed through a multiple-choice response item. In concrete terms, the question is “Do you experience pain frequently?” to which participants have to select one of the following choices: no, once a month, several times a month, several times per week or every day. The next question elicits information about how long the participant has been experiencing pain, in an open format.

The intensity, location and type of pain was assessed through the questionnaire PainDETECT (PD-Q)^[41]^ in its original version in English and its adaptation to the Spanish language.^[42]^ This questionnaire assesses the neuropathic component of conditions accompanied by chronic pain by means of a user-friendly self-administered form that does not require the presence of a professional. It is made up of 4 blocks: block 1 comprises 3 visual analogue scales (VAS) with 11 possible scores, in which 0 is equivalent to “absence of pain” and 10 to “maximum pain.” These 3 items explore pain at the present moment, more intense pain over the last 4 weeks, and mean pain over the last 4 weeks; block 2 presents 1 item with 4 possible answers consisting of images with their corresponding text, designed to define participants’ patterns of pain experienced over time. The options given are “persistent pain with slight fluctuations,” “persistent pain with pain attacks,” pain attacks with no pain between attacks’, and “pain attacks with pain between them”; block 3 consists of a drawing showing a front and rear view of a person, accompanied by 3 items asking about the location of pain and the direction in which it irradiates; block 4 is formed of 7 items in a 6-point Likert format that consult various characteristics of the pain experienced at the locations indicated, such as burning sensation, tingling or prickling sensation, pain from light chafing, sudden pain attacks, pain on contact with heat or cold, numbness and pain from slight pressure. Possible answers were “never,” “hardly noticed,” “slightly,” “moderate,” “strongly,” and “very strongly.”

In validating the original questionnaire, the authors obtained a significant bivariate correlation between items (*P* < .01) and an adequate internal consistency (Cronbach alpha = 0.83).^[41]^ The Spanish adaptation also presents good psychometric indices, with Cronbach alpha equal to 0.86 and the test-retest intraclass correlation to 0.93.^[42]^

#### 2.3.3. Pain appraisal.

To evaluate the variable “pain appraisal,” we used the questionnaire Pain Appraisal Inventory (PAI).^[43]^ This is one of the tools developed from the transactional model proposed by Lazarus and Folkman.^[16]^ It comprises 16 items with 6 answer choices (strongly disagree, moderately disagree, disagree mildly, mildly in agreement, moderately in agreement, strongly in agreement) that give shape to 2 factors: threat appraisal, referring to a negative interpretation of pain that is associated to unpleasant emotions and greater restrictions to activity, and challenge appraisal, relating to a more positive interpretation of experiencing pain that is linked to better quality of life.

With regard to psychometric qualities, the original questionnaire presents good internal consistency, with Cronbach alpha equal to 0.86 for the threat factor and 0.81 for the challenge factor. No Spanish version of this questionnaire was available, which led us to address its validation in Spanish language by means of the double inverse translation method. In the validated version, the threat factor scored a Cronbach alpha of 0.860, while the challenge factor equaled 0.864.

#### 2.3.4. Coping strategies.

The Questionnaire on Coping with Chronic Pain Revised (CAD-R)^[44]^ was applied to assessing the coping strategies used by participants experiencing pain. This is a reduced version of the Cuestionario de Afrontamiento al Dolor Crónico [questionnaire on coping with chronic pain] (CAD),^[45]^ and consists of 24 items with 5 response alternatives (never, rarely, sometimes, often, or always) that assess 6 factors: religion (cognitive or behavioral strategies stemming from religious or spiritual aspects), distraction (behaviors aiming to focus attention away from pain), mental self-control (cognitive efforts to diminish pain), self-affirmation (self-verbalization to improve one’s own mood), catharsis (seeking relief from pain through verbalization with other people), and information search (consultations conducted by the individual to gain further information on the problem find solutions).

The questionnaire presents good internal consistency indices, with Cronbach alpha scores in each of the factors of 0.94 for religion, 0.84 for catharsis, 0.75 for distraction, 0.80 for mental self-control, 0.77 for self-affirmation and 0.74 for information search. In this case, the questionnaire was not available in the English language, which prompted a translation likewise applying the double inverse translation method. In the English version, the McDonald omega indices obtained were 0.65 for distraction, 0.77 for information search, 0.93 for religion, 0.82 for catharsis, 0.80 for mental self-control, and 0.79 for self-affirmation.

#### 2.3.5. The interference of pain in everyday activities.

To evaluate the impact of pain in daily life a simple questionnaire was used, drawn up by the research team based on a review of the existing literature. This aim of this questionnaire is to assess the interference of pain in everyday tasks, specifically personal hygiene and autonomy, social and family life, job-related aspects, household chores, leisure and free time and sports and physical activity. It contains 6 items in VAS format on an 11 point scale, in which 0 represents “absence of interference” and 10 “maximum interference.” As for internal consistency indices, the Cronbach alpha score was 0.862.

#### 2.3.6. Quality of life.

Overall quality of life was evaluated by means of the questionnaire SF-12^[46]^ (Short Form-12 Health Survey) and its version in Spanish language.^[47]^ This is a reduced version of SF-36^[48]^ (Short Form-36 Health Survey), one of the most commonly used questionnaires in assessing quality of life linked to health. It comprises 12 Likert items aiming to assess the level of wellbeing and functional capacity in people over 14 years of age through 2 dimensions, namely physical health and mental health. Each dimension is evaluated on 6 items, whose answer format may comprise 2, 3, 5, or 6 choices. Scores under 50 indicate poorer health than the population mean, whereas scores above 50 indicate better health than the mean for the population of reference.

As for internal consistency indices, the English language version scores a Cronbach alpha of 0.89 in the physical health dimension and 0.76 for mental health. The Spanish version shows similar indices, with 0.85 for physical health and 0.78 for mental health.

### 2.4. Statistical analysis

Statistical analysis was performed using SPSS21.0 software. Data were expressed as percentages, means and standard deviations. Correlational analyses were performed using Pearson Correlation Analysis. Statistical significance was set at *P* < .05.

## 3. Results

Participants’ age varied between 18 and 85 years, with the mean at 41.20 years (standard deviation [SD] 13.81), and 75.6% of participants were women (n = 316) and 24.4% men (n = 102). A total of 36 nationalities were present in the sample, the most represented countries being Spain (28.2%, n = 118), United States of America (23.9%, n = 100) and United Kingdom (10.8%, n = 45). As for marital status, 44.5% were single, 38% married, 11.5% were in a civil partnership, 5% were divorced and 1% were widowed. Over half of the sample had a university degree (54.8%), 20% had vocational training, 15.6% secondary education, 3.3% primary education, 4.8% other studies and only 1.4% reported no official education. With reference to employment status, 51.2% of the sample were working at the time of answering the survey, 21.3% were unemployed, 10.8% were in the process of training, 6.7% were pensioners of working age, and 10% had retired. These data are shown in Table 1.

**Table 1:** Sociodemographic data in the full sample

Sociodemographic data (n = 418)		
**Gender and age**	Age	41.20 (18–85)
	Women (%)	316 (75.6)
	Men (%)	102 (24.4)
**Marital status**	Single (%)	186 (44.5)
	Married (%)	159 (38)
	Civil partnership(%)	48 (11.5)
	Divorced(%)	21 (5)
	Widowed(%)	4 (1)
**Education**	University Studies(%)	229 (54.8)
	Vocational training(%)	84 (20.1)
	Secondary Education(%)	65 (15.6)
	Primary Education(%)	14 (3.3)
	Other studies(%)	20 (4.8)
	Uneducated(%)	6 (1.4)
**Occupational status**	Working(%)	214 (51.2)
	Unemployed(%)	89 (21.3)
	Training(%)	45 (10.8)
	Pensioners (of working age)(%)	28 (6.7)
	Retired(%)	42 (10)

**n** = total number of participants in the sample under study.

According to clinical data (Table 2), 45.7% of the sample reported being affected by a mild condition, 40% a moderate condition and 14.3% a severe condition. OI type I was the most common, accounting for 45.5% of participants, followed by type III (20.8%), type IV (16.5%), type II (4.1%), and type V (1.7%), while 11.5% reported other types. It should nevertheless be pointed out that only 44% of the sample was in possession of a genetic study at the time of assessment. The approximate average number of fractures suffered by participants during the course of their lives was 62.62 (SD = 102), and the most common clinical signs were, in order of frequency, the presence of blue sclera (82.1%), short stature (66%), scoliosis (60.9%), hypermobility of the joints (56.3%) bone deformity (55.5%), vertebral collapse (43.7%), sprains (42.8%), dentinogenesis imperfecta (37.3%), eyesight problems (37%), and hearing difficulties (22.9%). Up to 45.9% were not habitual users of orthopedic aids, while 31.1% used wheelchairs, 10.5% crutches, 2.4% a walking frame, and the remaining 10% reported using other aids. A majority of the sample had not been in treatment with biphosphonates for the last 2 years (66.5%). Similarly, 76.3% of participants did not have regular physiotherapy appointments, 10.3% visited the physiotherapist approximately once a month, 3.6% twice a month and 9.8% at least once a week. Finally, regarding physical exercise, 41.9% reported normally taking no exercise, 22% exercised once a week, and 36.1% 2 or more days a week.

**Table 2:** Clinical data for the full sample.

Clinical data (n = 418)		
**OI severity**	Mild OI (%)	191 (45.7)
	Moderate OI (%)	167 (40)
	Severe OI (%)	102 (24.4)
**Type of OI**	Type I (%)	190 (45.5)
	Type II (%)	17 (4.1)
	Type III (%)	87 (20.8)
	Type IV (%)	69 (16.5)
	Type V (%)	7 (1.7)
	Other types (%)	48 (11.5)
**Clinical signs**	Approximate no. of fractures	62 (SD = 102)
	Blue sclera (%)	340 (81.3)
	Short stature (%)	276 (66)
	Scoliosis (%)	252 (60.3)
	Joint hypermobility (%)	233 (55.7)
	Bone deformities (%)	232 (55.5)
	Vertebral collapse (%)	181 (43.3)
	Frequent sprains (%)	179 (42.8)
	Dentinogenesis imperfecta (%)	156 (37.3)
	Eyesight problems (%)	153 (36.6)
	Hearing difficulties (%)	95 (22.7)
**Use of orthopedic aids**	None (%)	192 (45.9)
	Wheelchair (%)	130 (31.1)
	Crutches (%)	44 (10.5)
	Walking frame (%)	10 (2.4)
	Other aids (%)	42 (10)
**Treatment with biphosphonates in the last 2 years**	No treatment (%)	278 (66.5)
	With treatment (%)	140 (33.5)
**Do you visit the physiotherapist regularly?**	No (%)	319 (76.3)
	Once a month (%)	43 (10.3)
	Twice a month (%)	15 (3.6)
	At least once a week (%)	41 (9.8)
**Do you take regular exercise?**	No (%)	175 (41.9)
	Once a week (%)	92 (22)
	Two days or more per week (%)	151 (36.1)

**n** = total number of participants in the sample under study, **no**.= number, **oi** = osteogenesis imperfect.

**Figure 1.**
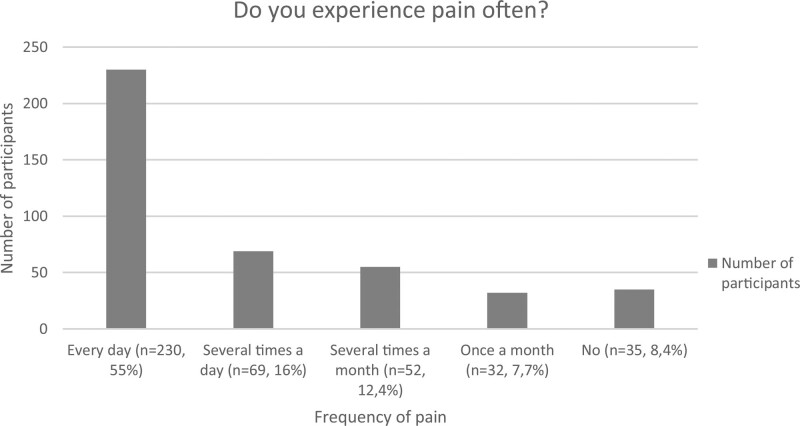
Pain frequency in the full sample (number of participants and % of the sample).

Pain was found to be notably present in the sample assessed, appearing daily in 55% of participants, several times a week in 16.5% and several times a month in 12.4%. A mere 7.7% reported experiencing pain around once a month and 8.4% reported not usually experiencing pain (Fig. 1). The pain lasted more than 6 months in 97% of the sample. Pain intensity was assessed by means of the 3 VAS scales present in the PD-Q questionnaire, with scores from 0 to 10. Mean pain intensity at the time of assessment was 4.67 (SD = 2.31), 6.94 (SD = 2.49) for the most intense pain over the last 4 weeks and 5.15 (SD = 2.260) for mean pain intensity over the last month (Table 3). These scores are affected by the responses given by participants who do not normally experience pain. If the above are excluded (16.1% of the sample), the mean scores, in the same order as above, are 5.15 (SD = 2.096), 7.57 (SD = 1.91), and 5.63 (SD = 1.2).

**Table 3:** Pain intensity measured with PainDETECT questionnaire: mean and standard deviation in the full sample and frequent pain sample.

	Full sample (n = 418)		Frequent pain sample (n = 351)	
	Mean	Standard deviation	Mean	Standard deviation
**Pain intensity at the present moment**	4.67	2.31	5.15	2.96
**Most intense pain over the last 4 weeks**	6.94	2.49	7.57	1.91
**Mean pain intensity over the last 4 weeks**	5.15	2.26	5.63	1.2

**n** = total number of participants in the sample under study.

The location of pain varied greatly, the back being the area most frequently affected (59.8% of the sample), followed by the pelvis (22.2%), lower limbs (22.2%), joints (9.6%), shoulders (9.1%), knees (8.6%), upper limbs (6.5%), neck (6.2%), and feet (5.3%). The results of the PD-Q suggest that 61.7% of the sample experience nociceptive pain, 16.3% neuropathic pain and 22% are doubtful.

To measure participants’ self-assessment of their own pain the PAI questionnaire was employed, which yields results on a scale of 1 through 6, on which a score of 3 or above indicate a significant presence of the studied variable. It was observed that the perception of pain as a threatening event, with a mean of 3.93 (SD = 1.16), was greater than when perceived as a challenge (2.87, SD = 1.19) (Table 4). On excluding from the analysis the individuals who do not or who rarely experience pain, both means increase, reaching 4.04 (SD = 1.1) for threat perception and 2.92 (SD = 1.17) for challenge appraisal.

**Table 4:** Pain appraisal measured with PAI questionnaire: mean and standard deviation in full sample and frequent pain sample.

	Full sample (n = 418)		Frequent pain sample (n = 351)	
	Mean	Standard deviation	Mean	Standard deviation
**Threat appraisal**	3.93	1.16	4,04	1.1
**Challenge appraisal**	2.87	1.19	2.92	1.17

**n** = total number of participants in the sample under study.

Regarding the coping strategies used to address pain, the most common was self-affirmation (14.29, SD = 3.73), followed by distraction (11.45, SD = 3.17), information search (10.77, SD = 3.99), mental self-control (10.03, SD = 3.91), catharsis (9.89, SD = 4.1), and religion (7.99, SD = 5.03) (Table 5).

**Table 5:** Strategies for coping with pain measured with CAD-R questionnaire: mean and standard deviation in full sample.

	Coping strategies	
	Mean	Standard deviation
**Self-affirmation**	14.29	3.73
**Distraction**	11.45	3.17
**Information search**	10.77	3.99
**Mental self-control**	10.03	3.91
**Catharsis**	9.89	4.1
**Religion**	7.99	5.03

CAD-R = Questionnaire on Coping with Chronic Pain Revised.

Pain was also found to interfere with the performance of everyday activities, principally affecting sports and physical activity, with a mean of 6.49 (SD = 3.12). In descending order, pain interfered in the performance of household tasks (.), in the workplace (5.35 SD = 3.32), in leisure activities (5.32, 2.93), in family and social relations (4.91, SD = 2.97), and in personal hygiene and autonomy (3.67, SD = 3.1) (Table 6).

**Table 6:** The interference of pain in everyday activities questionnaire designed by the authors and quality of life measured with SF-12 questionnaire: mean and standard deviation in full sample.

		Mean	Standard deviation
**The interference of pain in everyday activities**	**Personal hygiene and autonomy**	3.67	3.1
	**Family and social life**	4.91	2.97
	**Household tasks**	5.65	2.89
	**Job-related activity**	5.35	3.32
	**Sports and physical activity**	6.49	3.12
	**Leisure activities**	5.32	2.93
**Quality of life**	**Physical health**	36.25	25.76
	**Mental health**	54.06	25.68

**SF-12** = The 12-Item Short Form Health Survey.

Lastly, the SF-12 questionnaire was used to assess quality of life. Mean physical health index was 36.25 (SD = 25.76), while the value for mental health was 54.06 (SD = 25.68). Bearing in mind that the population mean stands at 50 (SD = 10) for both indices, we may deduce that perceived physical health is affected while mental health appears to be spared.

Below are some important links established between pain and sociodemographic and clinical variables (Tables 7 and 8). Firstly, pain was found to be more frequent in women than in men (*P* = .030), although with a small effect size (*R* = 0.11). A positive correlation was also found with age, the frequency of pain rising with participants’ age (*R* = 0.224***)

**Table 7:** Mean difference in pain frequency related to gender and clinical signs in OI.

			Pain frequency		
			Mean	*P*	Effect size
**Gender**		**Women**	3.1	.030*	*R* = 0.11
		**Men**	2.78		
**Clinical signs**	**Vertebral collapse**	**Yes**	3.2	.020*	*R* = 0.11
		**No**	2.91		
	**Sprains**	**Yes**	3.25	.015*	*R* = 0.12
		**No**	2.85		

* *P* ≤ .05;

**P ≤ .01;

***P ≤ .001.

**p** = probability value, **oi** = osteogenesis imperfecta, **r** = effect size of Pearson r correlation.

**Table 8:** Correlations between pain frequency and clinical data, sociodemographic data and quality of life.

		Pain frequency
		r
	**No. of fractures**	0.224***
**Clinical and sociodemographic data**	**Age**	0.175***
**Quality of life**	**Physical health**	−0.27***
	**Mental health**	−0.222***

*P ≤ .05;

**P ≤ .01;

*** *P* ≤ .001.

**r** = Pearson correlation coefficient.

No significant correlations were found with the degree of severity of the pathology, with the use of biphosphonates as medical treatment in the last 2 years, or with healthy habits such as physical exercise and physiotherapy. However, a positive correlation was observed with the number of fractures suffered in the life of each participant (*R* = 0.175***), and with clinical signs such as vertebral collapse (*P* = .020, ES = 0.11) and the presence of frequent sprains

Finally, the frequency of pain also sets up a significant positive correlation with quality of life, regarding both physical health (r = −0.270***) and mental health (r = −0.222***).

The intensity of pain was measured on 3 VAS scales assessing pain at the present moment, the most intense pain over the last 4 weeks and the mean intensity of pain in the last month. These 3 scales showed positive correlations with the number of fractures suffered in the life of each participant, as shown in Table 9, and only mean pain intensity showed a significant correlation to the severity of the condition (*R* = 0.113, *P* = .021). As before, no significant correlations were observed with taking physical exercise, receiving physiotherapy or being in treatment with biphosphonates.

**Table 9:** Correlations between pain intensity and clinical data.

	Pain intensity at the present moment	Most intense pain over the last 4 weeks	Mean pain intensity over the last 4 weeks
	r	r	r
**No. of fractures**	0.098*	0.138**	0.122*
**Severity of OI**	0.082	0.083	0.107*

* *P* ≤ .05;

** *P* ≤ .01;

***P ≤ .001.

**no**.= number, **r** = Pearson correlation coefficient.

Pain intensity was also found to be linked to the interference of pain in everyday activities. Thus, the 3 intensity indices correlated to all the areas assessed, as shown in Table 10. Likewise, a significant positive correlation was observed between the 3 pain intensity scales and physical and mental health. These results are also shown in Table 10.

**Table 10:** Correlations between pain intensity and the interference of pain in everyday activities and quality of life.

		Pain intensity at the present moment	Most intense pain over the last 4 weeks	Mean pain intensity over the last 4 weeks
**Interference of pain**		**r**	**r**	**r**
	**Personal hygiene and autonomy**	0.336***	0.342***	0.363***
	**Social and family life**	0.426***	0.424***	0.428***
	**Household tasks**	0.356***	0.382***	0.361***
	**Job-related activity**	0.329***	0.38***	0.354***
	**Sports and physical activity**	0.271***	0.338***	0.292***
	**Leisure activities**	0.322***	0.359***	0.342***
**Quality of life**	**Physical health**	−0.315***	0.351***	−0.365***
	**Mental health**	−0.276***	0.249***	−0.259***

*** *P* ≤ .001.

**r** = Pearson correlation coefficient.

Participants’ self-assessment of pain was divided into 2 indices: threat appraisal and challenge appraisal. The threat appraisal index established significant relationships with several variables (Table 11). First, a positive correlation was observed with the 3 items assessing pain intensity, that is, the intensity of pain at the present moment (*R* = 0.305, *P* < .000), the most intense pain experienced over the last 4 weeks (*R* = 0.285, *P* < .000) and mean pain intensity throughout the last month (*R* = 0.312, *P* < .000). The appraisal of pain as a threatening event also correlates to the interference of pain in everyday activities. Thus, the greater the perceived threat, the more pain interfered in personal hygiene and autonomy (*R* = 0.285, *P* < .000), family and social relations (*R* = 0.341, *P* < .000), job-related aspects (*R* = 0.198, *P* < .000), household tasks (*R* = 0.275, *P* < .000), sports and physical activity (*R* = 0.224, *P* < .000), and leisure activities (*R* = 0.296, *P* < .000). Finally, a negative correlation was also noted between threat appraisal and quality of life, both regarding physical health (r = −0.175, *P* < .000) and mental health (r = −230, *P* < .000). The appraisal of pain as a challenge, however, gave rise to no significant relationships with any of the variables described.

**Table 11:** Correlations between pain appraisal and its intensity, its interference in everyday activities and quality of life.

		Threat appraisal
		r
**Pain intensity**	**Pain intensity at the present moment**	0.305***
	**Most intense pain over the last 4 weeks**	0.285***
	**Mean pain intensity over the last 4 weeks**	0.312***
**Interference of pain**	**Personal hygiene and autonomy**	0.285***
	**Social and family life**	0.341***
	**Household tasks**	0.275***
	**Job-related activity**	0.198***
	**Sports and physical activity**	0.224***
	**Leisure activities**	0.296***
**Quality of life**	**Physical health**	−0.175***
	**Mental health**	−0.23***

*** *P* ≤ .001.

**r** = Pearson correlation coefficient.

Both types of appraisal were also found to correlate to the use of different coping strategies, as shown in Table 12.

**Table 12:** Correlation between pain appraisal and coping strategies.

		Threat appraisal	Challenge appraisal
		r	r
**Coping strategies**	**Distraction**	0.001	0.27***
	**Information search**	0.264***	0.039
	**Mental self-control**	0.229***	0.247***
	**Self-affirmation**	0.13**	0.407***
	**Religion**	0.161***	0.2***
	**Catharsis**	0.231***	0.103*

*** *P* ≤ .001.

**r** = Pearson correlation coefficient.

The various coping strategies, in turn, established certain significant correlations with the interference of pain in daily activities and quality of life, as shown in Table 13. Nevertheless, these relationships were generally of low intensity.

**Table 13:** Correlations between coping strategies and the interference of pain in everyday activities and quality of life

	Interference of pain					
	Personal hygiene and autonomy	Social and family life	Job-related activity	Household tasks	Sports and physical activity	Leisure activities
	r	r	r	r	r	r
**Distraction**	0.085	0.085	0.0795	0.094	0.032	0.082
**Information search**	0.111*	0.025	−0.05	−0.005	−0.097*	−0.053
**Mental self-control**	0.144**	0.100*	0.063	0.021	0.032	0.013
**Self-affirmation**	0.047	0.143**	0.092	0.026	0.015	0.011
**Religion**	0.1582**	0.191**	0.01	0.049	0.02	0.011
**Catharsis**	0.137**	0.102*	0.036	0.039	0.04	0.027
		Quality of life				
		Mental health			Physical health	
		r			r	
	**Distraction**	0.1			0.06	
	**Information search**	0.12			0.003	
	**Mental self-control**	−0.143**			−0.155**	
	**Self-affirmation**	0.026			0.034	
	**Religion**	0.015			0.02	
	**Catharsis**	0.047			−0.049	

* *P* ≤ .05;

** *P* ≤ .01.

**r** = Pearson correlation coefficient.

A significant relationship of a positive nature was observed between quality of life and the interference of pain in everyday activities. In sum, in all the areas assessed, as pain interference increased participants’ mental and physical health significantly declined. These results are displayed in Table 14.

**Table 14:** Correlation between pain interference in everyday activities and quality of life.

	Quality of life	
	Physical health	Mental health
	r	r
**Personal hygiene and autonomy**	−0.258**	−0.233**
**Social and family life**	−0.324**	−0.316**
**Job-related activity**	−0.287**	−0.301**
**Household tasks**	−0.342**	−0.270**
**Sports and physical activity**	−0.260**	−0.274**
**Leisure activities**	−0.310**	−0.289**

** *P* ≤ .01.

**r** = Pearson correlation coefficient.

## 4. Discussion

Chronic pain appears with high frequency in the adult population with OI, interferes in their everyday activities and affects their quality of life, especially their physical health. Participant’s own pain appraisal is significantly linked to their experience of pain and its consequences.

Up to 55% of the sample reported experiencing pain every day, 16% several times a week and 12.4% several times per month; hence, up to 83.4% of participants (n = 351) described having pain assiduously, with a duration >6 months in most cases. These data match those of other similar studies. For example, Balkefors et al^[12]^ found that 25 of the 29 participants in their sample reported frequent pain, whereas in the study by Arponen et al,^[11]^ 87% of participants reported experiencing pain every day. Despite the difficulty in establishing comparisons with the general population owing to the variety of nationalities taking part in this study, it is interesting to note that in Spain and in the United States of America, the 2 most represented countries (52% of the sample), the prevalence of chronic pain is 17.6% and 20.4%, respectively.^[49,50]^

Pain was found to be significantly more frequent in women than in men, although the differences were slight, and with a small effect size. Pain intensity and frequency were also found to increase with age, and with the accumulated number of fractures suffered during the patients’ lifetime. However, no significant correlations were observed between the frequency and intensity of pain and aspects such as the severity of the pathology, treatment with biphosphonates in the last 2 years, physiotherapy care or engaging in physical exercise. These data are similar to those found in other studies. For instance, Arponen et al,^[11]^ also found no correlation between the severity of the condition and the presence of pain. Similarly, Nghiem et al,^[13]^ describe how an accumulation of fractures during patients’ lives may cause the onset of chronic pain, and indicate the persistence of pain despite receiving pharmacological and nonpharmacological treatment. In any case, the benefits of physiotherapy and treatment with biphosphonates on chronic pain are evident,^[7,8,51,52]^ and the absence of correlation observed in this study may be related to its design, descriptive-correlational rather than experimental.

Participants described how their everyday activities were hindered by pain, the most affected being sports and physical activity, followed by household tasks, job-related activity, leisure activities, family and social relations, and personal autonomy. This had an impact on their quality of life, significantly reducing their mental and physical health in proportion to the level of interference. At all events, the results indicate a reduced physical health index, while mental health scores remain within healthy limits. Other studies assessing this topic have drawn similar conclusions. In a meta-analysis of quality of life in OI, Dahan-Oliel et al,^[33]^ point out that pain and the limitation and restriction of activities are associated to lower levels of physical health, which is poorer than that of the general population, whereas mental health stays at levels similar to the population mean. Similarly, in the study by Balkefors et al,^[12]^ on quality of life in OI, the authors highlight the strong correlation between physical limitations to everyday activities such as climbing stairs or going for walks, and physical health, which is also poorer than that found in the population of reference. However, as seen in this study, mental health seems to remain unaffected, while participants report high levels of life satisfaction.

Pain intensity was shown to be relevant to the performance of everyday activities in all spheres, generating greater interference the more intense the pain. A significant worsening was also observed in quality of life, regarding both mental and physical health, as pain frequency and intensity increased. In this regard, comparable results were found in the study by De Andrés et al,^[31]^ on health appraisal and coping strategies for tackling pain in a sample of pain tratment unit patients, in which participants with higher levels of pain reported greater interference in daily activities and worse mental and physical health status.

Generally speaking, participants in this study perceived pain as a threat, while pain viewed as a challenge obtained lower scores. In concrete terms, the mean for the variable “threat appraisal” was 3.93, more than 87% of the sample obtaining a score of 3 or above. This is important given that, as pointed out by the author of the questionnaire,^[43]^ scores >3 begin to indicate a notable threat component in appraisals of experiencing pain, which may impact the interference of pain on day to day responsibilities and functions.

In fact, the study revealed a significant link between pain appraisal as a threat and the interference of pain in everyday activities. The greater the perception of pain as a threatening event, the greater the prejudice to everyday activities. A similar effect was found with regard to quality of life, mental, and physical health worsening with a threatening appraisal of pain. In addition, a significant correlation was observed with pain intensity, which was heightened in individuals experiencing pain as threatening. This set of relationships between appraisal and other pain-related variables has been described in studies conducted on other populations. In their article reviewing the biopsychosocial model approach to pain, Gatchel et al,^[20]^ dedicate a section to this matter, stating that the appraisal of pain as a threat may lead to an increased perception of pain intensity and to the avoidance of daily activities likely to be interpreted as potentially hurtful. Likewise, Soriano and Monsalve^[53]^ in their study “appraisal, coping and emotion in patients with chronic pain” describe how participants with higher threat appraisal levels reported experiencing more intense pain and greater limitations to their everyday tasks than those with lower levels.

Pain appraisal as a threatening event, however, did not lead to establishing significant relationships with other variables pointing to better adjustment, as might have been expected. Nonetheless, the author of the questionnaire used found that, despite the appraisal of pain as a threat being associated with the pain’s intensity, this was not the case when pain was appraised as a challenge.^[43]^ She also described the profile for those inclined to address pain as a challenge as younger individuals, with fewer responsibilities (single, students, or part-time workers or childless). In our case, a significant negative correlation was found with age, although this was fairly small (r = −0.130, *P* = .008). Occupational or marital status showed no significant correlations.

Several studies have described the relationship between pain appraisal and coping strategies, as per the transactional model proposed by Lazarus and Folkman.^[16]^ For example, Ramírez-Maestre et al,^[24]^ in a sample of 122 patients with musculoskeletal pain, observed that high degrees of threat appraisal were linked to greater use of passive coping strategies and fewer active strategies, while the opposite occurred when the perception of pain as a challenge prevailed. Similar results are described in the meta-analysis of pain appraisal in Jackson et al.^[22]^ These results are not replicated herein. Threat appraisal, therefore, correlates both to active coping strategies (information search, mental self-control and self-affirmation) and passive coping strategies (religion and catharsis). The same applies to the appraisal of pain as a challenge, although in this case, while no correlation is established to the information search strategy, it does correlate to the distraction strategy. Furthermore, the correlation to self-affirmation strategies consisting in the capacity to maintain a positive frame of mind and self-motivation, is the strongest of all, this strategy being linked to lower psychological distress from pain.^[22,54]^

In general, coping strategies for tackling pain did not appear to establish significant links with the quality of life index, as has already been observed in other research studies.^[26]^ The mental self-control strategy alone is negatively linked to physical and mental health, but this correlation was slight. This relationship had already been described in another study by Monsalve, Soriano, and De Andrés^[55]^ in which the authors found that mental self-control worked negatively on patients’ overall health. Nonetheless, they also noted a positive correlation between the self-affirmation strategy and quality of life, an aspect that was not detected in the analysis presented herein.

## 5. Conclusions and limitations to this study

This study considers that chronic pain is a frequent reality among adults with OI, and is present in over 83% of the sample. Furthermore, this has an impact on everyday activities, causing greater interference as pain intensity increases. This eventually takes a toll on quality of life, which is impaired both regarding physical and mental health.

The way in which individuals rate their pain seems to influence how they experience it, a significant relationship being found between the perception of pain as a threatening event and increased pain intensity. Similarly, interference in everyday activities and loss of quality of life is seen to increase as the threatening nature of pain becomes more pronounced.

Finally, pain appraisal seems to carry more weight than coping strategies in the impact of pain on participants’ lives. This suggests that measures to manage cognitions of pain may bear more favorable outcomes than those focusing on modifying and learning coping strategies.

This study is not free from limitations. First, data were collected through an online survey. Although this method is being used with increasing frequency^[56]^ and offers advantages such as allowing access to extensive samples and reaching more disperse populations,^[57,58]^ its drawbacks include lack of control over participants and accessibility difficulties for individuals less familiar with new technologies, which may generate a bias in the sample.^[59]^ Second, some relevant psychological variables that would lead to a better understanding of the experience of pain have not been evaluated. Some examples of these are emotional responses such as anxiety and depression, indicators used in other studies to measure individuals’ adaptability to chronic pain.^[54]^ The decision to dismiss the inclusion of other variables in the study was taken in order to avoid fatigue bias, which may cause loss of attention and uniform, inadequate answers in excessively lengthy questionnaires.^[60]^ The correlational-descriptive design of the study does not allow causality relations to be established in the correlations described, despite which we have sought to back up observations with scientific evidence found in similar research works. Finally, it was not assessed whether during the month prior to completing the survey participants suffered a bone fracture. Although in adulthood patients do not show a high frequency,^[63]^ it is possible that the presence of a fracture would imply a higher score on the item “more intense pain over the last 4 weeks.”

To finish, it should be mentioned that this study addresses the status of pain in the adult population with OI from a general and exclusively descriptive perspective. This work is intended as an initial approach, to which further relevant relationships observed in the most representative samples may be added in future articles.

## Acknowledgments

We are grateful to all the adults with OI who have participated in this study. We are especially grateful to the Fundación AHUCE for funding this project, and to its director Julia Piniella for her support and participation in the conception and organization of the research. We also thank the University of Valencia for its collaboration, as well as the AHUCE, AMOI OIFE, Brittle Bone Society, OIF and many others for their help in disseminating the online questionnaire.

## Author contributions

Conceived and designed the analysis: Rubén Muñoz Cortés, José Francisco Soriano Pastor, Vicente Monsalve Dolz

**Collected the data:** Rubén Muñoz Cortés

**Contributed data or analysis tools:** Rubén Muñoz Cortés, José Francisco Soriano Pastor, Vicente Monsalve Dolz

**Performed the analysis:** Rubén Muñoz Cortés

**Wrote the paper:** Rubén Muñoz Cortés

**Other contribution (supervision of data analysis and study text):** José Francisco Soriano Pastor and Vicente Monsalve Dolz
